# Multiomic Analysis of Cereblon Expression and Its Prognostic Value in Kidney Renal Clear Cell Carcinoma, Lung Adenocarcinoma, and Skin Cutaneous Melanoma

**DOI:** 10.3390/jpm11040263

**Published:** 2021-04-01

**Authors:** Hyo Jae Shin, Kyung Jin Lee, Minchan Gil

**Affiliations:** 1 Department of Biological Sciences, Konkuk University, 120 Neungdong-ro, Gwangjin-gu, Seoul 05029, Korea; shinhyojae0606@gmail.com; 2 Department of Convergence Medicine, Asan Institute for Life Sciences, University of Ulsan College of Medicine, Asan Medical Center, Seoul 05505, Korea; 3 Department of Life Science, Hanyang University, Seoul 04763, Korea; 4 Department of Stem Cell and Regenerative Biotechnology, Konkuk University, 120 Neungdong-ro, Gwangjin-gu, Seoul 05029, Korea

**Keywords:** cereblon, multiomic analysis, lung adenocarcinoma, kidney renal clear cell carcinoma, skin cutaneous melanoma, prognostic value

## Abstract

Cereblon (CRBN) is a component of the E3 ubiquitin ligase complex that plays crucial roles in various cellular processes. However, no systematic studies on the expression and functions of *CRBN* in solid tumors have been conducted to date. Here, we analyzed *CRBN* expression and its clinical value using several bioinformatic databases. *CRBN* mRNA expression was downregulated in various cancer types compared to normal cells. Survival analysis demonstrated that overall survival was significantly positively correlated with *CRBN* expression in some cancer types including lung adenocarcinoma (LUAD), kidney renal clear cell carcinoma (KIRC), and skin cutaneous melanoma (SKCM). *CRBN* expression was downregulated regardless of clinicopathological characteristics in LUAD and KIRC. Analysis of genes that are commonly correlated with *CRBN* expression among KIRC, LUAD, and SKCM samples elucidated the potential CRBN-associated mechanisms of cancer progression. Overall, this study revealed the prognostic value of CRBN and its potential associated mechanisms, which may facilitate the development of anti-cancer therapeutic agents.

## 1. Introduction

Cancer is among the leading causes of human mortality worldwide, with 1,762,450 cases and 606,880 cancer-related deaths recorded in 2019 in the United States alone [1]. Cancer results from an accumulation of genetic aberrations in cells due to genetic predisposition or environmental oncogenic factors [2,3]. Several of these factors––smoking, alcohol consumption, diet, lack of exercise, viral infection, and exposure to environmental carcinogens and radiation––are associated with cancer incidence and progression [4,5,6,7,8]. Despite the recent progress in treatment over the past few decades, cancer remains a serious threat to human health. Therefore, the identification of differentially expressed genes with prognostic values is critical for the development of novel strategies for cancer diagnosis and treatment to enhance survival rates.

The cereblon protein, which is encoded by the *CRBN* gene, was first identified as a potential genetic factor that contributes to learning and memory [9]. However, *CRBN* expression was then identified in several other tissues and was found to play multiple roles associated with crucial biological functions, including cell metabolism and neural function [10,11]. *CRBN* dysregulation has been linked to several human diseases such as teratogenicity, leukemia, myeloma, mental retardation, and organ failure [12]. Cereblon is a component of the E3 ubiquitin ligase complex along with the damaged DNA binding protein 1 (DDB1) and Cullin-4A (CUL4A), where it acts as a substrate receptor for the targeted protein proteolyzed by the proteasome complex [13]. Immunomodulatory drugs bind to cereblon and modulate its proteolytic activity. For example, thalidomide inhibits ubiquitin ligase activity by binding to cereblon, which is the cause of its known teratogenicity [14]. Immunomodulatory drugs including thalidomide, pomalidomide, and lenalidomide have anti-proliferative activities in myeloma cells and induce T cell cytokine production [15]. *CRBN* expression in myeloma is also positively correlated with the survival of thalidomide-treated patients [16]. However, the expression and role of cereblon in tumorigenicity and tumor progression in a variety of cancer types has not been systematically studied.

Furthermore, cereblon binds and inhibits the activation of AMP-activated protein kinase (AMPK) by interacting with the α1 subunit of AMPK [17]. Physiologically, AMPK is a major metabolic regulator that preserves energy balance during metabolic stress [18]. In turn, *CRBN* deficiencies enhance AMPK activity in the lungs and suppress diabetic phenotypes in mice [19]. Moreover, depletion of *CRBN* suppresses the expression of inflammatory cytokines by enhancing AMPK and homooxygenase-1 activity in retinal cells and macrophages, as well as in a systemic inflammation mouse model [20,21]. Additionally, CRBN functions as a negative regulator of autophagy activation [22]. 

In the present study, we systematically characterized the expression of *CRBN* in various cancer types by analyzing publicly available expression datasets using web-based mining tools. *CRBN* expression was found to be significantly reduced in cancer tissues compared to normal tissues and was positively correlated with patient survival in multiple types of cancers including kidney renal clear cell carcinoma, lung adenocarcinoma, and skin cutaneous melanoma. Moreover, *CRBN*-associated pathways were identified through ontology and pathway analysis with co-related genes in multiple types of cancers. Our findings suggest the value of cereblon as a prognostic marker and potential drug target for different types of cancers from hematopoietic tumors.

## 2. Materials and Methods

### 2.1. CRBN mRNA Expression Analysis in Different Types of Cancer and Normal Tissues

*CRBN* expression levels in multiple types of cancer versus normal tissues were investigated using the Tumor Immune Estimation Resource (TIMER) (https://cistrome.shinyapps.io/timer/, accessed on 28 July 2020) and the Gene Expression Database of Normal and Tumor Tissues 2 (GENT2) (http://gent2.appex.kr/gent2/, accessed on 28 July 2020). In the DiffExp module of the TIMER web tool, *CRBN* expression between various types of tumor and normal tissues was obtained from RNA-seq data in The Cancer Genome Atlas (TCGA) database [23,24]. The expression levels were visualized as boxplots and statistically significant differences between each type of cancer and normal tissues were evaluated via the Wilcoxon test [25]. In the GENT2 database, the tissue-wide gene expression profile of CRBN was retrieved from multiple integrated Affymetrix U133Plus2 microarray datasets [26]. The statistical difference was evaluated between each type of cancer and normal tissue by a two-sample T-test. A *p*-value of less than 0.05 was considered statistically significant.

### 2.2. Patient Survival Analysis Related to CRBN Expression in Different Cancer Types

Kaplan–Meier survival analyses were carried out to compare two patient groups split by CRBN expression using the Kaplan–Meier plotter (https://kmplot.com/analysis/, accessed on 1 August 2020) and R2: Kaplan Meier Scanner (https://hgserver1.amc.nl/cgi-bin/r2/main.cgi, accessed on 1 August 2020) web tools. The plotter web tool enables the analysis of patient survival based on expression data from 54,000 genes in 21 different cancer types including 6234 breast cancer samples, 2190 ovarian cancer samples, 3452 lung cancer samples, and 1440 gastric cancer samples [27]. Overall survival (OS) was analyzed in breast and lung cancer patients with the “autoselect best cutoff” option for patient splitting using GeneChip microarray datasets in the Kaplan–Meier plotter web tool. The R2: Kaplan Meier Scanner was used to determine the OS of two patients groups split by CRBN expression in 515 lung adenocarcinoma (LUAD) patients, 533 kidney renal clear cell carcinoma (KIRC) patients, 468 skin cutaneous melanoma (SKCM) patients, 520 head and neck squamous cell carcinoma (HNSC) patients, 146 pancreatic adenocarcinoma (PAAD) patients, and 408 bladder urothelial carcinoma (BLCA) patients in the TCGA database. In all, survival analysis with the Kaplan–Meier plotter and R2: Kaplan Meier Scanner, *p*-values were calculated by the log–rank test between the two patient groups. OS was analyzed in KIRC and LUAD patients in each clinicopathological subsets based on the hazard ratio (HR) and log–rank *p*-values obtained from the Kaplan–Meier plotter: pan-cancer RNA-seq module. A *p*-value of less than 0.05 was considered statistically significant.

### 2.3. Gene Expression Analysis Based on Each Clinical Characteristic Factor

*CRBN* expression boxplots according to various clinicopathological factors were generated using the UALCAN web tool with the default settings (http://ualcan.path.uab.edu/index.html, accessed on 23 August 2020) [28]. Specifically, *CRBN* expression levels were analyzed against normal kidney tissues according to the clinical characteristics of KIRC patients including individual cancer stage, race, gender, age, tumor grade, histological subtype, and nodal metastasis status. Expression of *CRBN* in LUAD was also analyzed in patient groups sorted by the same aforementioned clinical characteristics, albeit with the inclusion of smoking habits and TP53 mutation status. Statistical differences between patient groups were estimated by Student’s t-test and summarized with *p*-values in Supplementary Materials Table S2. A *p*-value of less than 0.05 was considered statistically significant.

### 2.4. Protein Expression Analysis in Various Types of Cancer

Protein expression in each type of cancer and normal tissue was obtained using mass-spectrometry-based proteomic data from the Clinical Proteomic Tumor Analysis Consortium CPTAC Confirmatory/Discovery cohorts through the UALCAN website [29].

### 2.5. Analysis of Correlated Genes with CRBN Expression

The KIRC, LUAD, and SKCM datasets from the TCGA database were analyzed using the R2: Genomics Analysis and Visualization Platform (https://hgserver1.amc.nl/cgi-bin/r2/main.cgi, accessed on 19 August 2020) to identify genes the expression of which were correlated with that of CRBN. The list of correlated genes were obtained with significance of Pearson correlation adjusted by a false discovery rate. Commonly correlated genes among the KIRC, LUAD, and SKCM datasets were identified by creating a Venn diagram of the top 500 most highly negatively and positively correlated genes in each type of cancer using the GeneVenn website (http://genevenn.sourceforge.net/, accessed on 19 August 2020) [30]. Associated pathways and gene ontology were analyzed with a list of commonly correlated genes using the Enrichr web tool (https://amp.pharm.mssm.edu/Enrichr/, accessed on 19 August 2020) [31].

## 3. Results

### 3.1. CRBN mRNA Expression in Various Human Cancer Types and Normal Tissues

**Figure 1:**
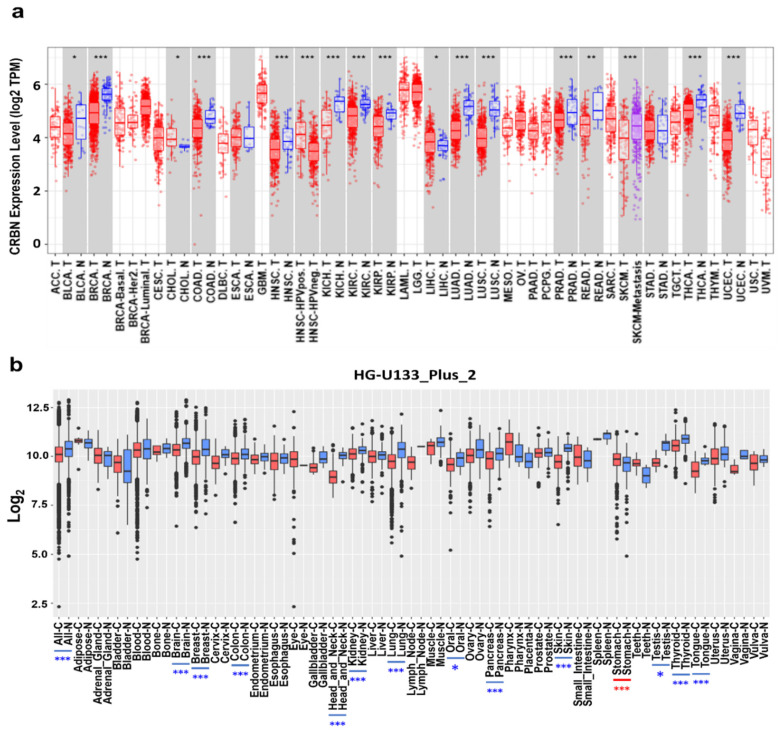
*CRBN* mRNA expression in various cancer types and corresponding normal tissues. (**a**) *CRBN* expression in different types of human cancer and normal tissues was determined from RNA-sequencing data from the Cancer Genome Atlas (TCGA) database using the Tumor IMmune Estimation Resource (TIMER) web tool (https://cistrome.shinyapps.io/timer/, accessed on 28 July 2020). Significant differences between each type of tumor and normal tissues were marked with asterisks (* *p* < 0.05, ** *p* < 0.01, *** *p* < 0.001). Abbreviations of cancer types are listed in Supplementary Materials Table S2. The letters “T” and “N” followed by the tumor type abbreviation indicate “tumor” and “normal,” respectively. (**b**) mRNA expression patterns of CRBN across various types of tumor and normal tissues were retrieved from the Gene Expression Database of Normal and Tumor Tissues 2 (GENT2) (http://gent2.appex.kr/gent2/, accessed on 28 July 2020). Boxplots were generated to illustrate the median and the 25th and 75th percentiles; the dots represent the outliers. Red boxplots represent cancer samples, and blue boxplots represent normal samples. Significant differences between each type of tumor and its normal counterpart are marked by blue asterisks (higher expression in normal tissue) or red asterisks (higher expression in the tumor).

CRBN mRNA expression was identified in different cancer types and normal tissue pairs using the TIMER and GENT2 databases. CRBN mRNA transcription levels were significantly downregulated in 13 out of 16 cancer types compared to normal tissues (Figure 1a). Nonetheless, two types of cancer exhibited CRBN upregulation, namely cholangiocarcinoma (CHOL) and liver hepatocellular carcinoma (LIHC) (Figure 1a). Afterward, after examining microarray data in the GENT2 database using the HG-U1333_Plus_2 platform, we found that CRBN mRNA expression was significantly downregulated in some cancer types such as brain, breast, colon, head and neck, kidney, lung, pancreas, skin, thyroid, and tongue (Figure 1b). Only stomach cancer exhibited CRBN mRNA upregulation compared to normal stomach tissues. However, CRBN mRNA expression was generally downregulated in multiple types of cancer compared to their corresponding normal tissues.

### 3.2. Correlation Between Survival and CRBN Expression in Various Types of Cancer

**Figure 2:**
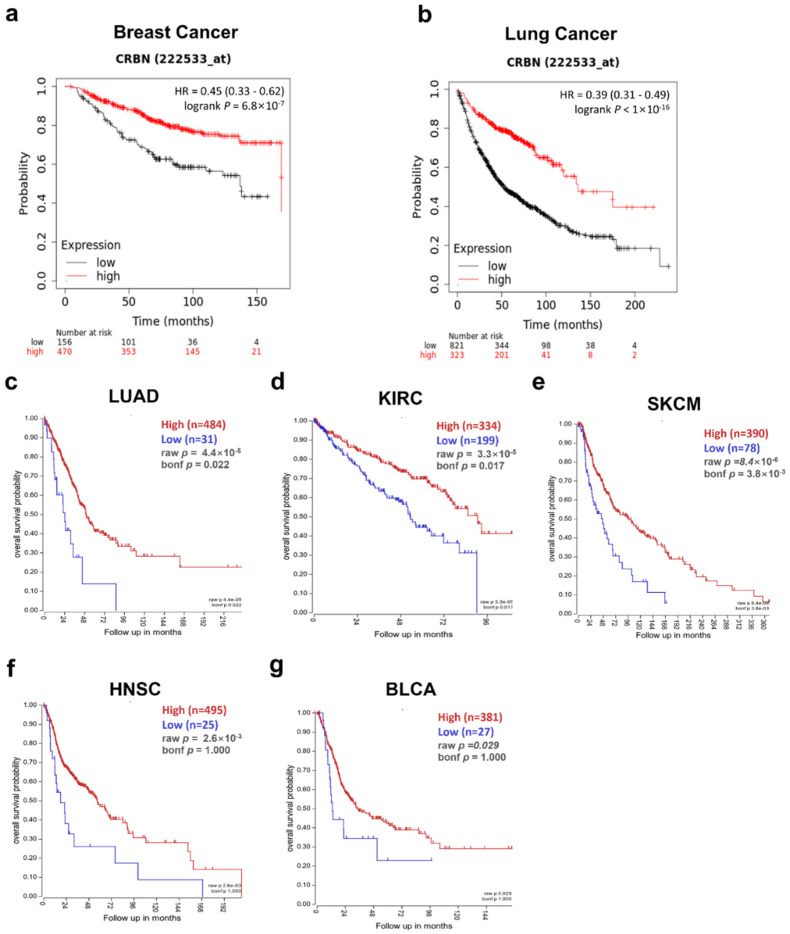
Correlation of CRBN expression with patient overall survival (OS) in various cancer types. OS was compared between the high expression and low expression groups using Kaplan–Meier curves. These survival curves were retrieved from the Kaplan–Meier plotter database for (**a**) breast cancer (n = 1764) and (**b**) lung cancer (n = 1144) or from the R2: platform with TCGA datasets for (**c**) lung adenocarcinoma (LUAD; n = 515), (**d**) kidney renal clear cell carcinoma (KIRC; n = 533), (**e**) skin cutaneous melanoma (SKCM; n = 468), (**f**) head neck squamous cell carcinoma (HNSC; n = 520), and (**g**) bladder urothelial carcinoma (BLCA; n = 408). All patient groups were split to minimize their *p*-value.

The correlation between patient survival and *CRBN* expression was analyzed using KM plotter and the R2: Kaplan Meier Scanner. First, the OS of breast cancer and lung cancer patients were positively correlated with *CRBN* expression according to the microarray-based database of the KM plotter. Poorer OSs were associated with patient groups with low *CRBN* expression in breast cancer (HR = 0.45, *p* = 6.8 × 10^−0.7^; Figure 2a) and lung cancer (HR = 0.39, *p* < 1 × 10^−16^; Figure 2b). Next, we use the R2: Kaplan Meier Scanner with the TCGA datasets to identify cancer types in which lower *CRBN* expression was associated with decreased patient survival rates. Among the five cancer types with a significant positive correlation between patient survival and *CRBN* expression (Figure 2c–h), LUAD, KIRC, and SKCM exhibited the most significant differences in OS between the high and low expression groups. Furthermore, OS was analyzed using the hazard ratio (HR) according to clinicopathological factors in the KIRC and LUAD patient groups (Table 1, Kaplan–Meier plots shown in Supplementary Materials Figure S1). The KIRC patient subgroups with significant correlation between OS and *CRBN* expression were the stage 3 (HR = 0.38; 0.21–1.01, *p* = 0.00053), female (HR = 0.43; 0.26–0.71, *p* = 0.00083), male (HR = 0.61; 0.42–0.89, *p* = 0.0094), white (HR = 0.53; 0.39–0.74, *p* = 0.00013), grade 3 (HR = 0.54; 0.34–0.86, *p* = 0.0087), and low mutation burden (HR = 0.38; 0.17–0.83, *p* = 0.012) subsets. In LUAD, the stage 4 (HR = 0.15; 0.03–0.68, *p* = 0.0051), female (HR = 0.63; 0.41–0.98, *p* = 0.039), male (HR = 0.42; 0.27–0.65, *p* = 5.60 × 10^−0.5^), white (HR = 0.59; 0.42–0.83, *p* = 0.0023), and low mutation burden (HR = 0.42; 0.28–0.64, *p* = 2.60 × 10^−0.5^) patient subgroups exhibited a significant correlation between OS and *CRBN* expression. 

### 3.3. Changes in CRBN Expression Associated with Clinicopathological Factors in KIRC and LUAD Patients

**Figure 3:**
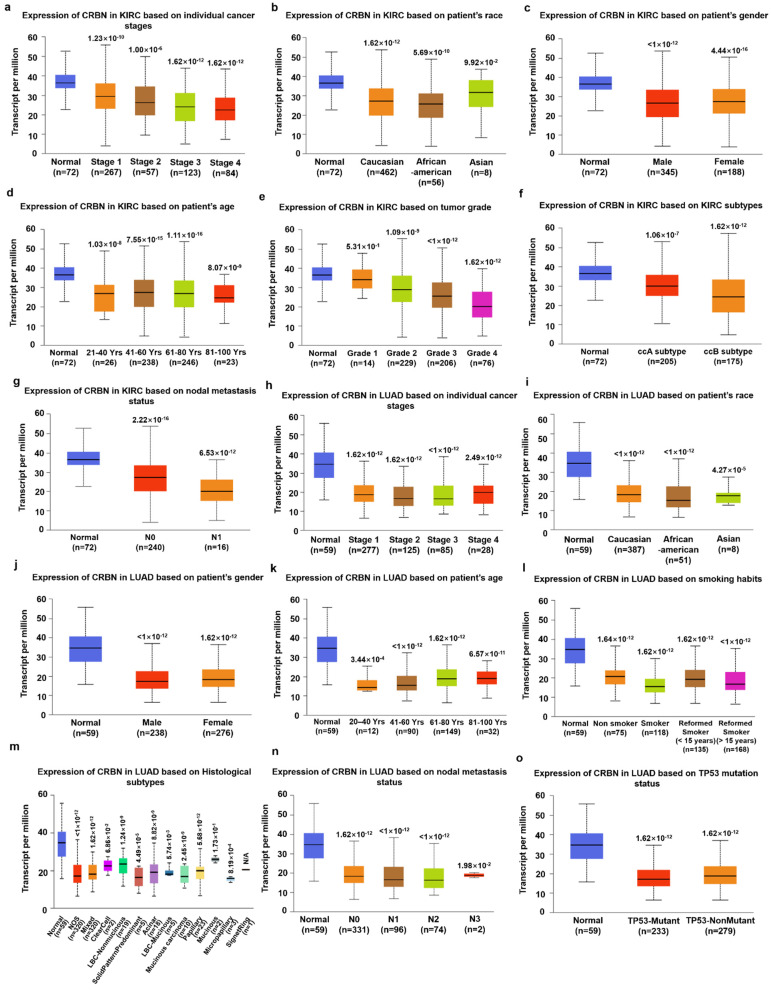
Differences in CRBN expression according to the clinicopathological factors of KIRC and LUAD patients. The expression levels of CRBN according to each clinicopathological characteristic were visualized with boxplots using the UALCAN web tool to explore the TCGA dataset (http://ualcan.path.uab.edu/index.html, accessed on 23 August 2020). Specifically, the mRNA expression of CRBN in KIRC was plotted against (**a**) individual cancer stages, (**b**) patient race, (**c**) patient gender, (**d**) patient age, (**e**) tumor grade, (**f**) KIRC subtypes, and (**g**) nodal metastasis status. Similarly, LUAD expression was plotted against (**h**) individual cancer stages, (**i**) patient race, (**j**) patient gender, (**k**) patient age, (**l**) smoking habits, (**m**) histological subtypes, (**n**) nodal metastasis status, and (**o**) TP53 mutation status.

Given the correlation between *CRBN* expression and KIRC, LUAD, and SKCM patient survival in the TCGA database, *CRBN* mRNA expression was analyzed according to numerous clinicopathological factors in KIRC, LUAD, and SKCM patients using the UALCAN analysis tools. Regardless of clinicopathological property subsets, *CRBN* expression was significantly lower in KIRC (Figure 3a–g). Interestingly, *CRBN* expression decreased according to cancer progression (stage 1, stage 2, stage 3, and stage 4, Supplementary Materials Table S1), tumor grade (grade 2, grade 3, and grade 4), and nodal metastasis status (N0 and N1) (Figure 3a,e,g). Based on KIRC subtype comparisons, *CRBN* expression in the ccB subtype was significantly lower than in the ccA subtype (Figure 3f). In LUAD, *CRBN* expression was also significantly lower than in normal tissues in most LUAD patient subsets divided by various clinicopathological characteristics, which generally did not affect *CRBN* expression except for smoking habits (Figure 3h–o). Specifically, smoking downregulated *CRBN* mRNA expression substantially more than any of the other analyzed factors (Figure 3l).

### 3.4. CRBN Protein Expression in Various Cancer Types

**Figure 4:**
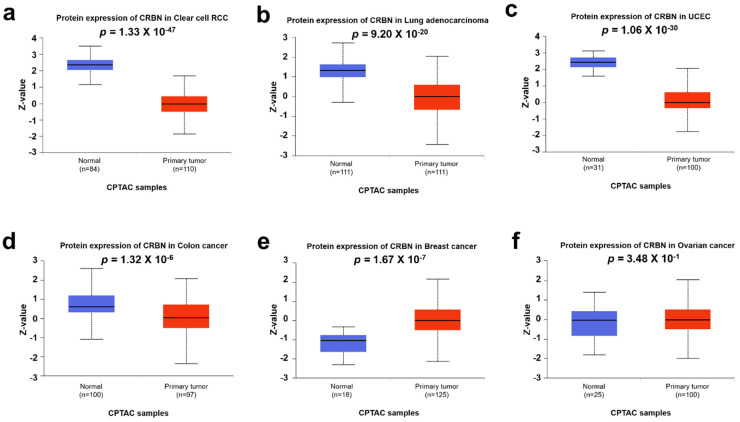
CRBN protein expression in various cancer types. CRBN expression in each type of cancer and normal tissues were explored in (**a**) clear cell renal cell carcinoma (RCC), (**b**) lung adenocarcinoma (LUAD), (**c**) uterine corpus endometrial carcinoma (UCEC), (**d**) colon cancer, (**e**) breast cancer, and (**f**) ovarian carcinoma based on the Clinical Proteomic Tumor Analysis Consortium (CPTAC) Confirmatory/Discovery dataset using the UALCAN web tool. Z-values represent standard deviations from the median across samples for each given cancer type.

Differential CRBN protein expression in cancer and normal tissues was examined using the UALCAN and human protein atlas websites. The protein expressions of CRBN in CPTAC samples were visualized with boxplots using the UALCAN web tool (Figure 4). Similar to the mRNA levels shown in Figure 1, CRBN protein expressions in clear cell renal cell carcinoma (RCC), lung adenocarcinoma, uterine corpus endometrial carcinoma (UCEC), and colon cancer were markedly downregulated compared to their normal tissue counterparts, which corresponded with their mRNA expression differences (Figure 4a,d). However, protein level expression was significantly higher in breast cancer cells compared to normal cells, which contradicted the mRNA level trends (Figure 4e). Moreover, CRBN protein expression in ovarian cancer was not significantly altered (Figure 4f), unlike mRNA expression.

### 3.5. Pathways and Gene Ontology Analysis of Commonly Correlated Genes with CRBN Expression in KIRC, LUAD, and SKCM

**Figure 5:**
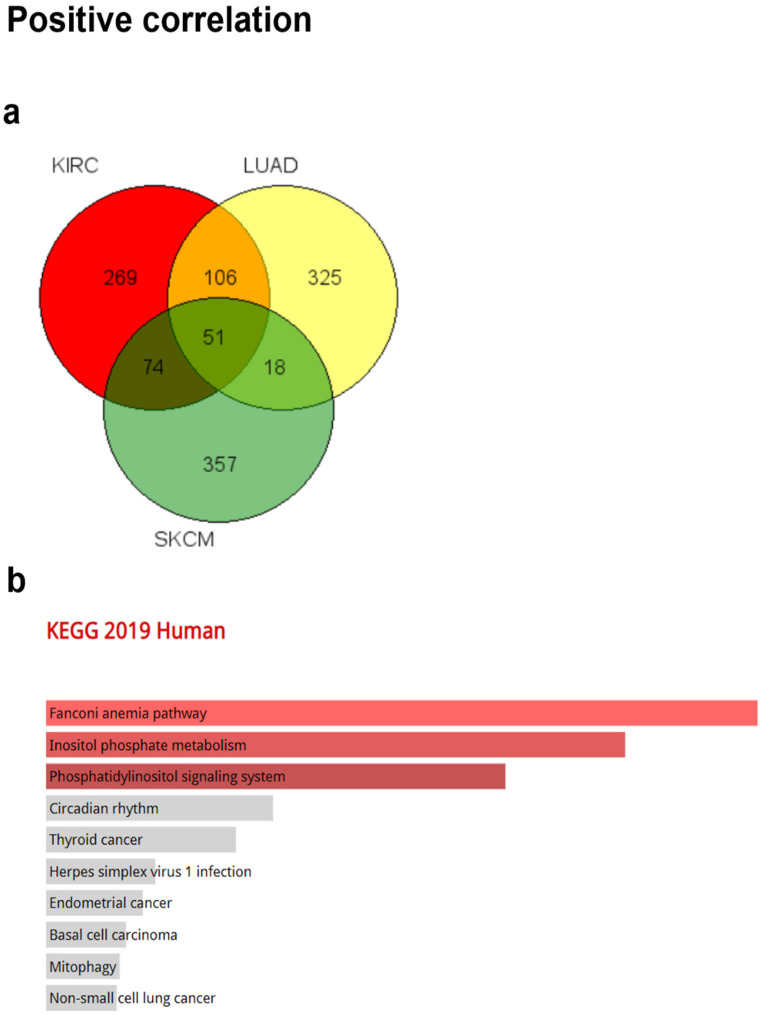

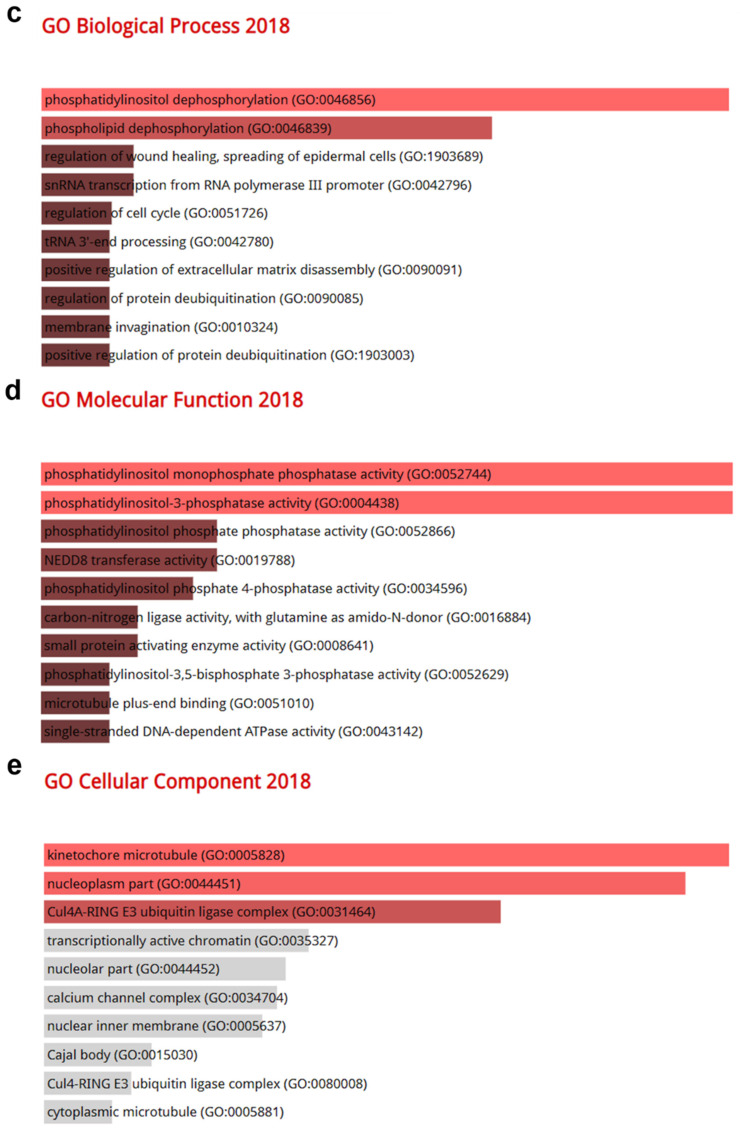

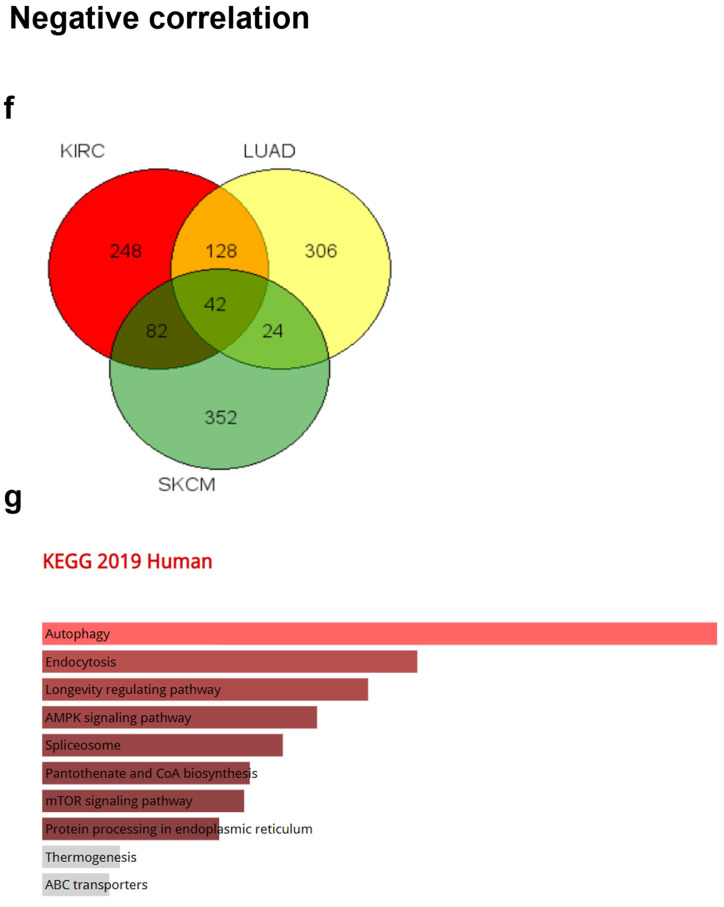

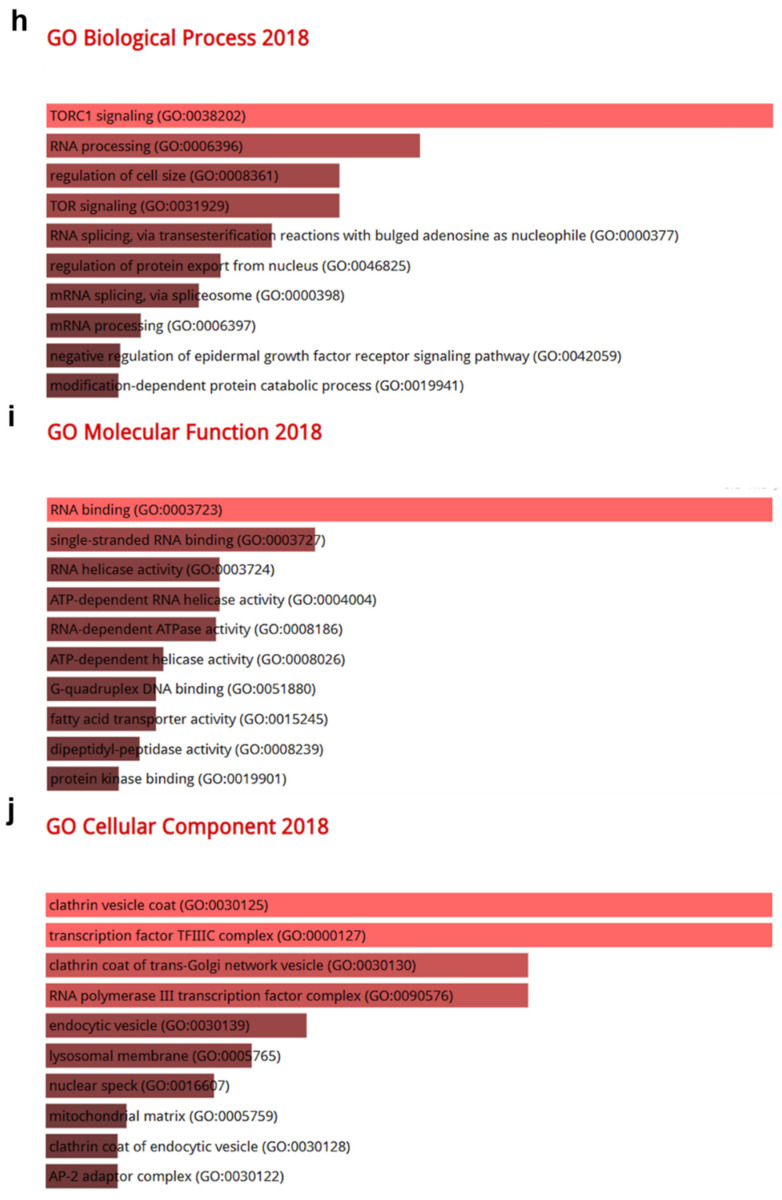
Gene Ontology (GO) and pathway analysis based on the genes that were co-altered with CRBN in KIRC, LUAD, and SKCM. The genes that were most strongly co-altered with CRBN in KIRC, LUAD, and SKCM were obtained using the R2: Genomics Analysis and Visualization Platform datasets (https://hgserver1.amc.nl/cgi-bin/r2/main.cgi, accessed on 19 August 2020). A list of genes that were co-altered with CRBN among the three types of cancer studied herein (KIRC, LUAD, and SKCM) were identified with a Venn diagram via the GeneVenn website (genevenn.sourceforge.net). Bar graphs were retrieved from the Enrichr website (http://amp.pharm.mssm.edu/Enrichr/, accessed on 19 August 2020) to illustrate the gene ontology (GO) and signaling pathways of common correlated genes. (**a**) Venn diagram of the positively correlated genes in the three types of cancer. (**b**) Kyoto Encyclopedia of Genes and Genomes (KEGG) pathways 2019. (**c**) Enrichment of GO Biological Process (2018). (**d**) Enrichment of GO Molecular Function (2018). (**e**) Enrichment of GO Cellular Component (2018). Similarly, (**f**) a Venn diagram was used to illustrate the negatively correlated genes in the three types of cancer. (**g**) KEGG pathways 2019. (**h**) enrichment of GO Biological Process (2018). (**i**) enrichment of GO Molecular Function (2018). (**j**) enrichment of GO Cellular Component (2018). The bar graphs represent the ratio of the percent composition based on the proteomic data vs. the percent composition based on the genome annotation. The length of the bar represents the significance of each specific gene set or term. Brighter colors indicate higher significance.

To identify signaling pathways associated with CRBN expression changes, ontology analysis was conducted using the top-500 most associated genes the expressions of which were co-altered with CRBN expression either positively or negatively in the KIRC, LUAD, and SKCM datasets of the TCGA database. The common positively and negatively correlated genes among the three cancer types were visualized using a Venn diagram (Figure 5a,f). A total of 51 genes that were positively correlated with CRBN expression were identified (Figure 5a) and subjected to an ontology analysis through the Enrichr website. The 10 top-ranked Kyoto Encyclopedia of Genes and Genomes (KEGG) pathways from these 51 positively correlated genes included the “Fanconi anemia pathway,” “inositol phosphate metabolism,” and “phosphatidylinositol signaling system” (Figure 5b). Moreover, according to GO analysis, the most significantly associated terms were “phosphatidylinositol dephosphorylation” in the “biological process” classification (Figure 5c), “phosphatidylinositol monophosphate phosphatase activity” in “molecular function” (Figure 5d), and “kinetochore microtubule” in “cellular component” (Figure 5e). 

Furthermore, a total of 42 genes that were negatively correlated with CRBN expression were identified between KIRC, LUAD, and SKCM (Figure 5f). The 10 top-ranked KEGG pathways associated with these 42 negatively correlated genes included “autophagy,” “endocytosis,” “longevity regulation pathway,” “AMPK signaling pathway,” “spliceosome,” “pantothenate and CoA biosynthesis,” “mTOR signaling pathway,” and “protein processing in endoplasmic reticulum” (Figure 5g). According to GO analysis, the most significantly related terms associated with the 42 negatively correlated genes were “TORC1 signaling” in “biological process” (Figure 5h), “RNA binding” in “molecular function” (Figure 5i), and “clathrin vesicle coat” in “cellular component” (Figure 5j).

## 4. Discussion

This study systematically assessed CRBN expression and its prognostic value in various cancer types using a variety of bioinformatic analysis tools. Most cancer types exhibited a lower CRBN expression compared to normal tissues. Particularly, the LUAD, KIRC, and SKCM patient groups with lower CRBN expression had poorer survival rates than those with higher CRBN expression.

In KIRC patients, CRBN mRNA expression was more downregulated in advanced cancer stages characterized by clinicopathologic factors such as grade, stage, and nodal metastasis. Tumors are defined by the appearance of tumor cells in a tissue, particularly by their degree of anaplasia, as determined by microscopic imaging [32,33]. In KIRC, a high tumor grade resulted in a markedly poorer OS than a low grade [34]. The prognostic stage of cancer is determined not by its microscopic morphology, but by the degree of its anatomic spreading, which is based on an evaluation of the tumor, regional lymph node, and metastasis (i.e., the so-called TNM stage) [32,35]. A longer TNM stage progression results in a lower 5-year survival rate in patients diagnosed with renal cell carcinoma [36]. CRBN expression in nodal metastasis level N1 (i.e., metastasis in one to three axillary lymph nodes) was lower than in N0 (i.e., no regional lymph node metastasis), as illustrated in Figure 3g [37]. Based on the KIRC subtype, the ccA subtype overexpresses a set of genes involved in hypoxia, angiogenesis, fatty acid metabolism, and organic acid metabolism, whereas the ccB subtype overexpresses genes involved in cell differentiation, epithelial to mesenchymal transition (EMT), cell cycle, and transforming growth factor beta (TGFβ), resulting in a more aggressive cancer progression [38]. In fact, KIRC subtype ccB has a worse survival outcome than ccA [39]. In our results, CRBN expression was also lower in the riskier ccB subtype than in the ccA subtype (Figure 3f). Overall, these results suggest that CRBN expression might be in volved in suppression of tumor progression in KIRC. In LUAD, cancerous tissues had less CRBN expression in smokers than nonsmokers or two reformed smoker groups (Figure 3I). Furthermore, the survival rates of non-smoking lung cancer patients were much better than those of smoking patients [40]. However, there were no significant differences in CRBN expression among cancer stages (Figure 3h) in LUAD although a higher risk of stage 2, 3, and 4 compared to stage 1 was evident in LUAD patients in TCGA database [41]. Despite clear differences in OS and CRBN expression between LUAD patient samples and the normal counterparts, there was no correlation of CRBN expression with OS in tumor stage progression. That might be related to a lower contribution of CRBN expression compared to other factors in LUAD cancer progression. CRBN expression in SKCM also had a positive overall prognostic value (Figure 2c). However, significant differences in CRBN expression among subgroups with different clinicopathological characteristics were not evident (data not shown). 

An analysis of the correlation between patient survival and gene expression in subgroups of clinicopathological characteristics provided insight into the specific subgroups in which CRBN expression could have clinical value as a prognostic marker or a therapeutic target. For example, in LUAD, CRBN expression had stronger correlation (*p* = 0.0051) with OS in the stage 4 subgroup of patients than in the other subgroups (Table 1) although there was no significant difference in expression among stages. Investigation of CRBN-expression phenotypes and related pathways in stage 4 cancer could reveal the role of CRBN in late stage of lung cancer and provide supportive evidences for the clinical use of CRBN and CRBN-related pathways as therapeutic target or prognostic marker for stage for patients.

Ontology analyses with 51 commonly positively correlated genes were conducted to elucidate the CRBN-associated pathways shared among KIRC, LUAD, and SKCM patients. Based on KEGG pathway analysis, the most highly associated pathway was the Fanconi anemia pathway. Particularly, the Fanconi anemia (FA) pathway is composed of 19 FA proteins and their associated proteins, all of which are associated with DNA interstrand crosslink repair [42]. Genetic inactivation of the FA pathway causes developmental defects, bone marrow failure, chromosome instability disorder, pancytopenia, and an increased risk of malignancies via the loss of several biological processes such as DNA repair and cell cycle progression [43,44,45,46,47,48]. The FA pathway is essential for tumor suppression via genome protection mechanisms [49]. Moreover, according to the “biological process” and “molecular function” terms of the GO analysis, phosphatidylinositol (PI) phosphatase was the most highly ranked pathway. PI phosphatases including PTEN are considered tumor suppressors because their activity inhibits the phosphoinositide-3 kinase (PI3K) pathway, which is associated with cellular transformation and cancer metastasis [50,51,52,53]. Therefore, the ontology terms and pathways associated with CRBN expression suggested the potential mechanisms by which CRBN expression suppresses cancer.

Pathways and ontology terms negatively correlated with CRBN expression were also analyzed using the 42 common genes that were found to be negatively correlated with CRBN in the three cancer types. Importantly, these genes that were negatively correlated with CRBN expression may negatively affect the prognoses of these cancer types. According to our KEGG pathway analyses, autophagy was the most highly associated pathway, which is required for tumor maintenance, tumor survival during environmental stress, and providing metabolic intermediates to highly resource-demanding cancer cells [54]. Moreover, in established tumors, autophagy promotes chemoresistance to anticancer therapies against metabolic stressors such as nutrient restriction, hypoxia, and absence of growth factors [55,56]. In addition to the autophagy pathway, other negatively correlated pathways such as AMPK signaling, mTOR signaling, and spliceosome are involved in tumor cell progression [57,58,59]. AMPK is closely involved in drug resistance by inducing autophagy and modulating cancer stem cells [60]. A previous study has already reported the modulation of AMPK signaling by CRBN [17]. Moreover, mTOR signaling plays a role in growth stimulation and cell cycle progression, and therefore its deregulation can lead to tumorigenesis, cell proliferation, angiogenesis, metastasis, and chemoresistance in various cancers including melanoma [58,61,62,63]. The analysis of pathways that are negatively correlated with CRBN expression suggested the potential cancer-promoting mechanisms of CRBN downregulation. Therefore, characterizing the common genes that are correlated with *CRBN* expression in KIRC, LUAD, and SKCM patients implied the potential pathways of *CRBN* in carcinogenesis and cancer progression.

## Figures and Tables

**Table 1:** Correlation between CRBN expression and prognosis in various clinicopathological subsets of KIRC and LUAD patients. Bold numbers indicates a statistically significant correlation with a *p*-value less than 0.05.

Clinicopathological Factors	KIRC			LUAD		
	N	Hazard Ratio	*p*-Value	N	Hazard Ratio	*p*-Value
**Stage**						
1	265	0.73 (0.41–1.33)	0.31	270	0.65 (0.4–1.07)	0.086
2	57	3.55 (0.95–13.23)	**0.046**	119	0.59 (0.34–1.02)	0.056
3	123	0.38 (0.21–0.67)	**0.00053**	81	0.63 (0.35–1.14)	0.12
4	82	0.61 (0.37–1.01)	0.053	26	0.15 (0.03–0.68)	**0.0051**
**Gender**						
Female	186	0.43 (0.26–0.71)	**0.00083**	270	0.63 (0.41–0.98)	**0.039**
Male	344	0.61 (0.42–0.89)	**0.0094**	234	0.42 (0.27–0.65)	**5.60 × 10^−0.5^**
**Race**						
White	459	0.53 (0.39–0.74)	**0.00013**	387	0.59 (0.42–0.83)	**0.0023**
Asian	8	-	-	8	-	-
Black/African American	56	0.67 (0.17–2.57)	0.55	52	0.5 (0.18–1.35)	0.16
**Grade**						
1	14	-	-	0	-	-
2	227	1.27 (0.7–2.33)	0.43	0	-	-
3	206	0.54 (0.34–0.86)	0.0087	0	-	-
4	75	1.37 (0.78–2.41)	0.27	0	-	-
**Mutation burden**						
high	168	0.61 (0.35–1.06)	0.074	255	0.72 (0.46–1.13)	0.15
low	164	0.38 (0.17–0.83)	**0.012**	244	0.42 (0.28–0.64)	**2.60 × 10^−0.5^**

## Data Availability

The data presented in this study are available on request from the corresponding author. The data are not publicly available due to privacy restrictions.

## Supplementary Materials

The following materials are available online at https://www.mdpi.com/article/10.3390/jpm11040263/s1, Figure S1: Correlation of CRBN expression to the KIRC and LUAD patient’s overall survival determined by each clinicopathological factor in Km plotter. Table S1: statistical significances for each intra-clinicopathological subfactor in the KIRC, LUAD, and SKCM datasets. Table S2: TCGA cancer type abbreviations.
