# Supplementary material for: Multiomic Analysis of Cereblon Expression and Its Prognostic Value in Kidney Renal Clear Cell Carcinoma, Lung Adenocarcinoma, and Skin Cutaneous Melanoma
Source: J Pers Med. 2021 Apr 1;11(4):263. doi: 10.3390/jpm11040263 (PMC8065640; doi:10.3390/jpm11040263)
Supplement: Supplementary file 1 [file jpm-11-00263-s001.pdf]

Supplementary Figure

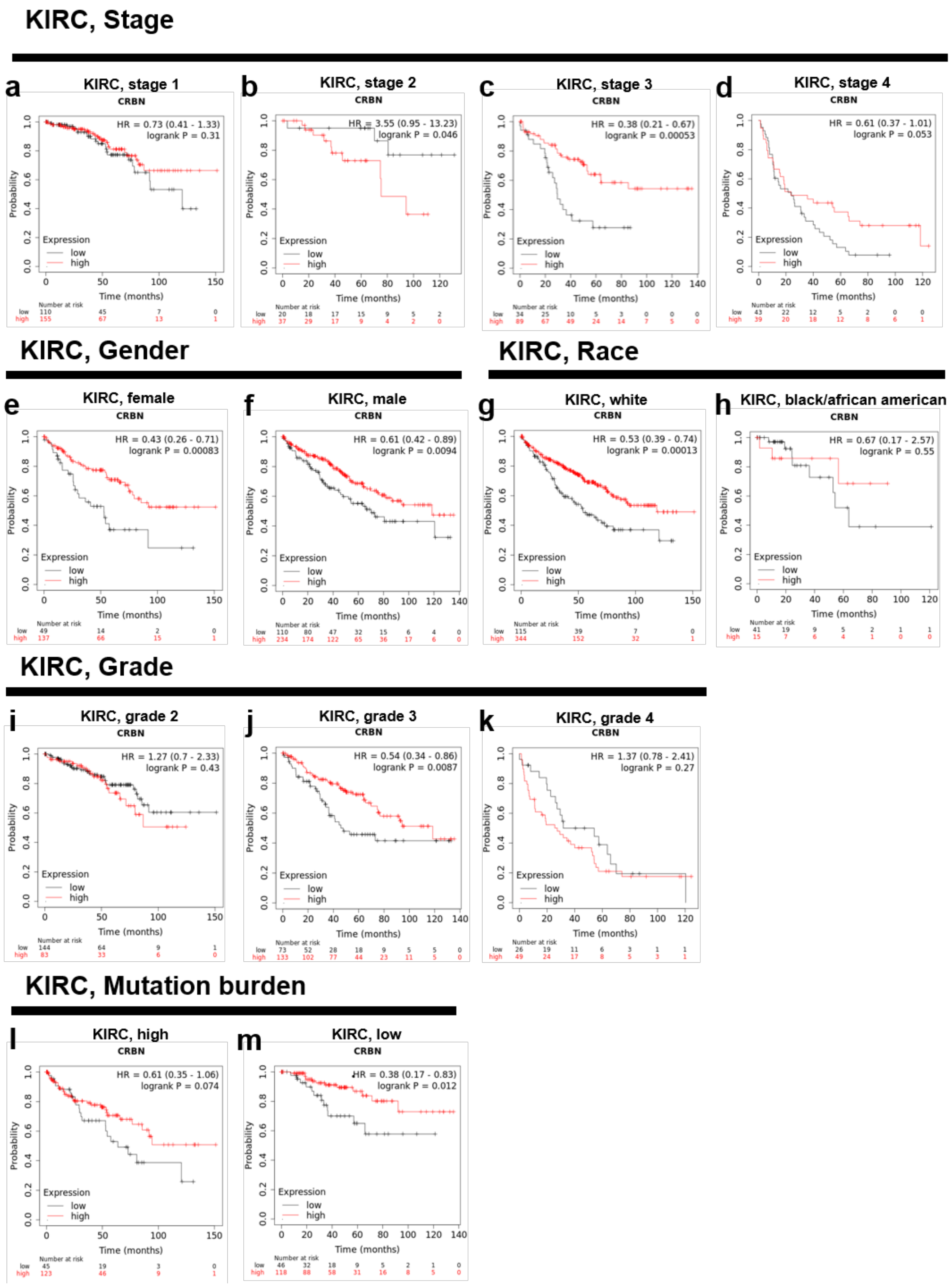

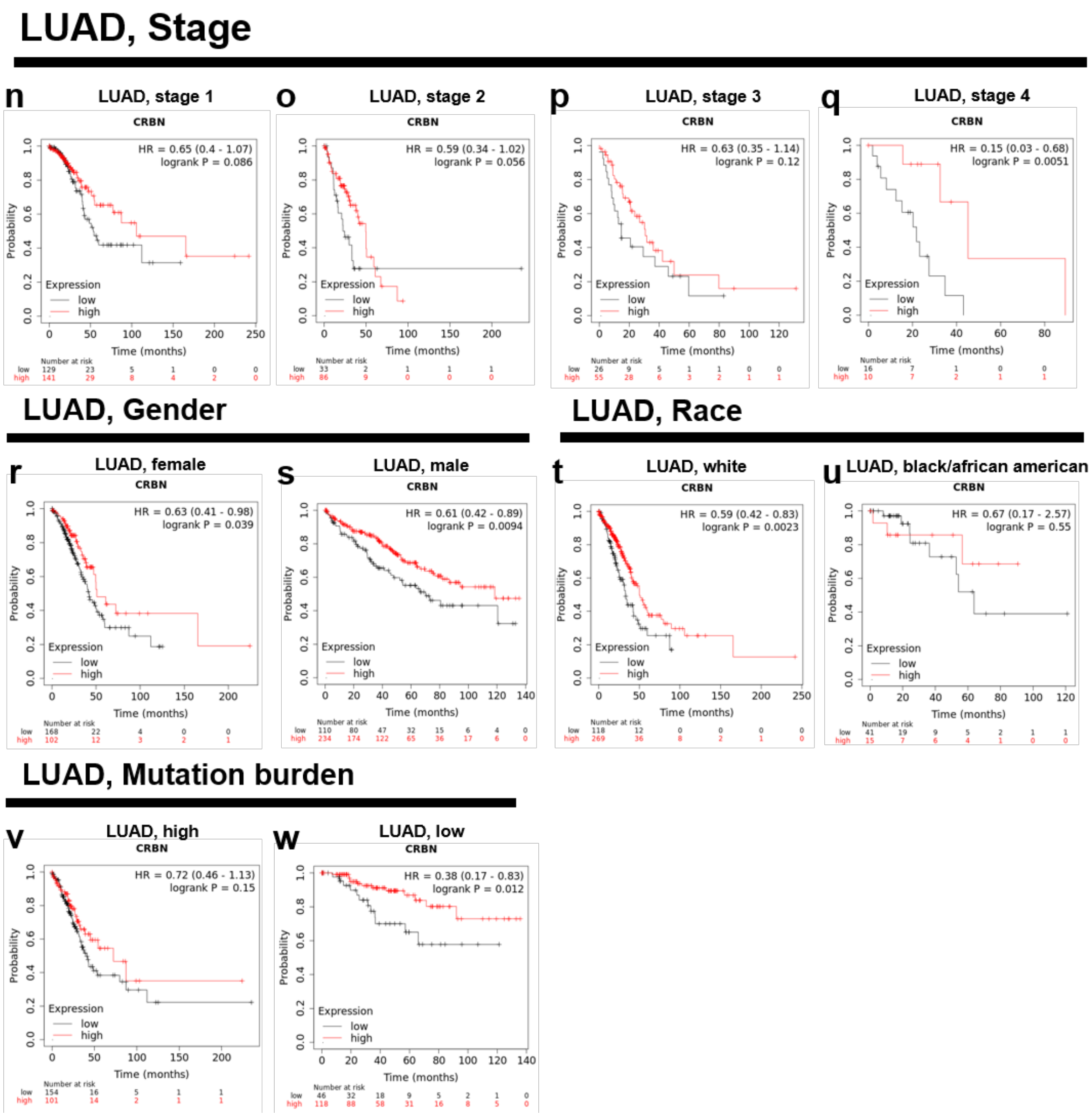

Supplementary figure S1. Correlation of CRBN expression to the KIRC and LUAD patient's overall survival determined by each clinicopathological factor in Km plotter. Overall survival about the high and low CRBN expression in kidney renal clear cell carcinoma (KIRC) patients restricted to subtypes of stage (a-d), gender (e-f), race (g-h), grade (i-k), and mutation burden (l-m). Likewise, prognostic relevance of CRBN in lung adenocarcinoma (LUAD) patients confined by stage (n-q), gender (r-s), race (t-u), and mutation burden (v-w).

Supplementary Tables

Supplementary table S1. Statistical significances between each intra-clinicopathological subfactor in KIRC, LUAD, and SKCM. *P<0.05, **P<0.01, ***P<0.001

Clinicopathological factor		p-value	
		KIRC	LUAD
**Cancer stage**			
Normal-vs-	stage 1	***	***
	stage 2	***	***
	stage 3	***	***
	stage 4	***	***
Stage 1-vs-	stage 2	2.57E-01	6.00E-02
	stage 3	***	2.05E-01
	stage 4	***	8.99E-01
stage 2-vs-	stage 3	*	7.61E-01
	stage 4	*	3.05E-01
stage 3-vs-	stage 4	6.58E-01	4.37E-01
**Tumor grade**			
Normal-vs-	grade 1	5.31E-01	-
	grade 2	***	-
	grade 3	***	-
	grade 4	***	-
Grade 1-vs-	grade 2	5.60E-02	-
	grade 3	***	-
	grade 4	***	-
Grade 2-vs-	grade 3	***	-
	grade 4	***	-
Grade 3-vs-	grade 4	***	-
**Nodal metastasis status**			
Normal-vs-	N0	***	***
	N1	***	***
	N2	-	***
	N3	-	*
N0-vs-	N1	*	1.19E-01
	N2	-	1.19E-01
	N3	-	8.59E-01
N1-vs-	N2	-	9.22E-01
	N3	-	9.40E-01

N2-vs-	N3	-	9.21E-01
**KIRC subtypes**			
Normal-vs-	ccA subtype	***	-
	ccB subtype	***	-
ccA subtype-vs-	ccB subtype	***	-
**Smoking habits**			
Normal-vs-	Non smoker	-	***
	Smoker	-	***
	Reformed smoker (<15 yrs)	-	***
	Reformed smoker (>15 yrs)	-	***
Non smoker-vs-	Smoker	-	***
	Reformed smoker (<15 yrs)	-	2.09E-01
	Reformed smoker (>15 yrs)	-	**
smoker-vs-	Reformed smoker (<15 yrs)	-	***
	Reformed smoker (>15 yrs)	-	5.10E-02
Reformed smoker (<15 years)-vs-	Reformed smoker (>15 yrs)	-	*

Supplementary table S2. Abbreviation of TCGA cancer types

Abbreviation	Type of Cancer
ACC	Adrenocortical carcinoma
BLCA	Bladder Urothelial Carcinoma
BRCA	Breast invasive carcinoma
CESC	Cervical squamous cell carcinoma and endocervical adenocarcinoma
CHOL	Cholangio carcinoma
COAD	Colon adenocarcinoma
DLBC	Lymphoid Neoplasm Diffuse Large B-cell Lymphoma
ESCA	Esophageal carcinoma
GBM	Glioblastoma multiforme
HNSC	Head and Neck squamous cell carcinoma
KICH	Kidney Chromophobe
KIRC	Kidney renal clear cell carcinoma
KIRP	Kidney renal papillary cell carcinoma
LAML	Acute Myeloid Leukemia
LGG	Brain Lower Grade Glioma
LIHC	Liver hepatocellular carcinoma
LUAD	Lung adenocarcinoma
LUSC	Lung squamous cell carcinoma
MESO	Mesothelioma

OV	Ovarian serous cystadenocarcinoma
PAAD	Pancreatic adenocarcinoma
PCPG	Pheochromocytoma and Paraganglioma
PRAD	Prostate adenocarcinoma
READ	Rectum adenocarcinoma
SARC	Sarcoma
SKCM	Skin Cutaneous Melanoma
STAD	Stomach adenocarcinoma
TGCT	Testicular Germ Cell Tumors
THCA	Thyroid carcinoma
THYM	Thymoma
UCEC	Uterine Corpus Endometrial Carcinoma
UCS	Uterine Carcinosarcoma
UVM	Uveal Melanoma

17
